# Phytophthora pluvialis *maintenance*, *spore production and detached needle assays*

**DOI:** 10.1371/journal.pone.0293817

**Published:** 2024-03-21

**Authors:** Sophie Eccersall, Leann S. Vinson, Rebecca McDougal, Claudia-Nicole Meisrimler

**Affiliations:** 1 School of Biological Science, University of Canterbury, Christchurch, New Zealand; 2 Scion (New Zealand Forest Research Institute, Ltd.), Rotorua, New Zealand; 3 Biomolecular Interaction Centre, University of Canterbury, Christchurch, New Zealand; University of Nebraska-Lincoln, UNITED STATES

## Abstract

*Phytophthora pluvialis* is an oomycete that primarily infects *Pinus radiata* and *Pseudotsuga menziesii* causing the destructive foliar disease red needle cast (RNC). Recent observations show that *P*. *pluvialis* can also infect western hemlock inducing resinous cankers. High-throughput and reproducible infection assays are integral to find key information on tree health and oomycete pathogenicity. In this protocol, we describe the propagation and spore induction of *P*. *pluvialis*, followed by detached needle assays for verification and quantification of virulence of *P*. *pluvialis* in *P*. *radiata* needles. These needle assays can be employed for high-throughput screening of tree needles with diverse genetic backgrounds. In downstream analysis, Quantitative PCR (qPCR) was utilized to assess relative gene expression, as exemplified by candidate RxLR effector protein PpR01. Additional techniques like RNA sequencing, metabolomics, and proteomics can be combined with needle assays and can offer comprehensive insights into *P*. *pluvialis* infection mechanisms.

## Introduction

*Phytophthora pluvialis* is an oomycete that primarily infects *Pinus radiata* and *Pseudotsuga menziesii* causing the destructive foliar disease red needle cast (RNC) [1]. RNC causes accelerated needle senescence and premature defoliation of trees and can lead to loss of growth. Typical disease symptoms include a dark resinous band, which extends to discoloured khaki lesions on the needle, followed by premature needle shedding. First recovered in 2002 in Oregon, *P*. *pluvialis* is found throughout the pacific northwest of the USA and New Zealand, and most recently the United Kingdom, causing branch and stem cankers and crown die back on mature western hemlock (*Tsuga heterophylla)* [2].

A reliable and reproducible protocol was created for the maintenance and growth of *P*. *pluvialis* and detached needle assays in laboratory setting. Detached needle assays have been performed on both known and unknown hosts of *P*. *pluvialis* and provide insight into virulence for breeding and research purposes, otherwise difficult to access due to the tree hosts slow growth and reproduction [3]. Detached needle assays can be combined with DNA, RNA, protein, and metabolite analysis [4] from infected and control tissues for further investigation of molecular mechanisms of the infection process. High throughput and reproducible infection assays are integral to find key information around tree health and oomycete pathogenicity. Classical or conventional breeding is one of the principal ways to improve pest and disease resistance. In tree species, not only is this costly, but is substantially complicated by their long generation times [5]. An -omics based approach can generate large amounts of data on forest tree resistance mechanisms which can ultimately contribute to the improvement and protection of commercially important trees, such as *Pinus radiata* by providing insight on effector targets and recognition [6]. This information mitigates the need for blind breeding which becomes costly and time consuming.

In this protocol, we describe the propagation and spore induction of *P*. *pluvialis*, followed by detached needle assay for conducting the virulence assay of *P*. *pluvialis* on *P*. *radiata* needles. These needle assays can be used for semi-high throughput assays to screen hundreds of trees genotypes in a laboratory setting and allow for further downstream analysis as shown by Graham et al., 2018 [5]. Additionally, we performed qPCR on *P*. *pluvialis* mycelium and both infected and non-infected *P*. *radiata* pine needles to confirm the presence or absence of oomycete RNA as shown in S1 [7].

*Phytophthora* species require cultivation on the appropriate selective media for successful isolation and investigation in laboratory conditions [8]. Important characteristics to observe are mycelium growth habit- aerial or appressed; mycelium pattern- uniform, radiate, stellate, or petaloid; and the structures present in agar—sporangia, oospores, hyphal swellings and chlamydospores [9]. Five New Zealand strains of *P*. *pluvialis* received from Scion (New Zealand Forest Research Institute, Ltd),were propagated in the laboratory at the University of Canterbury. In contrast to the original protocol for maintenance [1], *P*. *pluvialis* was grown on cV8 agar in this study [9]. All five strains grew on this medium similar to the original carrot agar medium with appressed hyphae with an angular and petaloid growth pattern. This phenotype confirmed previous descriptions of growth patterns for these *P*. *pluvialis* strains [10].

Zoospore production is a crucial part of the infection process of *Phytophthora* species [1, 11]. For needle infection assay experiments, a previous zoospore induction protocol was tested without success in reliable zoospore induction. To circumvent this issue, two main points of the original protocol were modified: 1) the agar and broth media for *P*. *pluvialis* growth 2) the treatments used to induce zoospores. Carrot agar and the carrot broth were replaced by 20% cV8 agar and broth, supplemented with β-sitosterol [6]. The cV8 medium is well-known media for growing *Phytophthora* species [12] and supports development of sporangia, making it an ideal media to optimise *P*. *pluvialis* growth. Within seven days, all five *P*. *pluvialis* strains grew without issues on the new media type and followed the appropriate growth patterns [9] and were ready for use in experiments. Upon induction of zoospores following the new protocol, the number of zoospores released increased significantly. Following the initial release, the zoospore solution was collected and used for needle infection assays. Once the zoospore solution was siphoned off, the petri dish containing the sporangia was flooded a second time with cold sterile pond water and repeated shock treatments. Upon this second induction treatment, a second “wave” of released zoospores was observed. This second wave of zoospores was also used to perform needle infection assays. No change of virulence between first and second wave of zoospores was noted. Plates containing mature sporangia can be re-induced multiple times over successive days.

## Material and methods

The protocol described in this peer-reviewed article is published on protocols.io dx.doi.org/10.17504/protocols.io.kqdg3p9d7l25/v1 and is included for printing as supporting information file 1 with this article.

### Expected results

**Fig 1:**
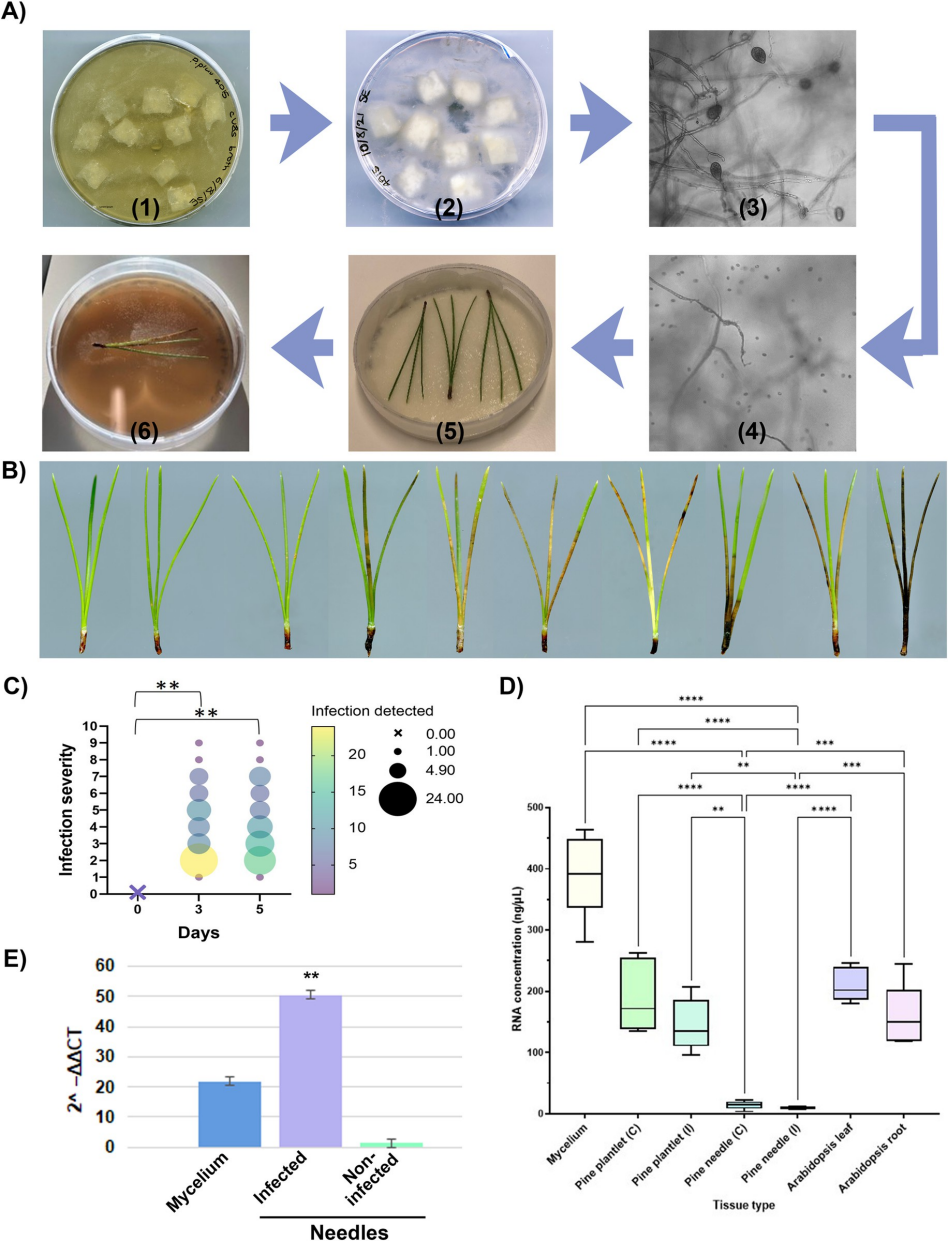
A) Protocol at a glance: (1) agar cubes in v8 broth; (2) agar cubes in sterilised pond water; (3) formation of mature sporangia; (4) zoospore induction and release; (5) detached needle assays; new mycelium propagation. B) Severity of infected pine samples from left to right on a scale of 1–10. C) Graph depicting infection severity (visual quantification) from detached needle assays on a scale of 1–10; n = 65 needles. KEY: Colour scale and circle size indicating the number of replicates and the level of infection; × indicates the full set of replicates at day zero with no infection symptoms. D) Graph depicting RNA concentrations from various tissue samples. C = control; (I) = infected. E) Graph depicting expression of PpR01 normalised to P. pluvialis actin-1 calculating its 2^–ΔΔCT.

Maintenance and spore production of *P*. *pluvialis* is essential for experiments associated to downstream infection assays to test for resistance or sensitivity in *Pinus radiata*, *Pseudotsuga menziesii*, *Tsuga heterophylla* and other potential host species. For quantitative infection assays numbers of zoospores will need to be the same, allowing comparison between infection severity. Generally, this protocol will allow for a minimum amount of 2.5 x 10^3^ mL of zoospores from the first wave of zoospore solution. Similar yields can be expected from subsequent waves by refreshing the petri dish with 15 mL of sterile pond water. For detached needle assays, the suggested number of spores/μl 2.5 x 10^3^. Although currently the minimum number of zoospores for successful infection is unknown, the suggested number will allow qualitative and quantitative infection of needles showing symptoms within three days. If the infection is successful, it is expected that 100% of needles show signs of infection at day three and day five post infection. In the shown example (Fig 1C.) a two-way ANOVA analysis was performed and demonstrated statistically significant difference in infection between day zero and day three and five respectively (p-value = 0.0072); no statistically significant difference was observed between day three and five (p-value = >0.999).

To demonstrate expected results for downstream analysis, RNA extraction was chosen. RNA extraction is essential for many applications, such as quantitative RT-PCR, RNA sequencing, Yeast-2-Hybrid cDNA library preparation. Analysis indicated no statistically significant difference between RNA concentrations extracted from control treated needles vs *P*. *pluvialis* infected pine needles (after three days); importantly, using the Plant/Fungi Total RNA purification kit (Norgen Biotek) to extract RNA from mycelium generally results in higher RNA amounts than pine samples. This needs to be considered during experimental planning. The pine needles used in the presented RNA extraction were ≤1 year old and harvested from trees 3 years of age or older. It can be stated that younger pine needles will result in higher amounts of RNA or protein than older pine needles (≥1 year). Additionally, for comparison, *Arabidopsis* leaves and roots have been extracted with the same RNA extraction protocol, indicating a significantly higher RNA concentration than for pine samples in general (Fig 1C and I; p-value≤0.0001). Furthermore, a statistically significant difference was observed for RNA extraction of pine plantlets vs needles for control and infected samples. These results are not unsurprising, as the phenolic compounds in mature pine needles can make RNA extraction difficult. Nevertheless, no effect is to be expected for RNA concentrations in dependence of the infection for day zero and day five. Importantly, longer infection time will lead to increased cell death resulting in reduced RNA and protein concentrations.

To confirm the presence of infection in the detached needle assay, quantitative RT-PCR was performed on three sample types. *P*. *pluvialis* mycelium, *P*. *radiata needles* infected with *P*. *pluvialis* zoospores and non-infected needles were tested for the expression of the candidate RxLR effector protein PpR01. No expression of PpR01 was detected in non-infected needles compared to high expression in mycelium and infected needles (Fig 1E).

## Supporting information

S1 File Step-by-step protocol, also available on protocols.io dx.doi.org/10.17504/protocols.io.kqdg3p9d7l25/v1. (PDF)
