# Supplementary material for: Phytophthora pluvialis maintenance, spore production and detached needle assays
Source: PLoS One. 2024 Mar 21;19(3):e0293817. doi: 10.1371/journal.pone.0293817 (PMC10956741; doi:10.1371/journal.pone.0293817)
Supplement: S1 File — (PDF) [file pone.0293817.s001.pdf]

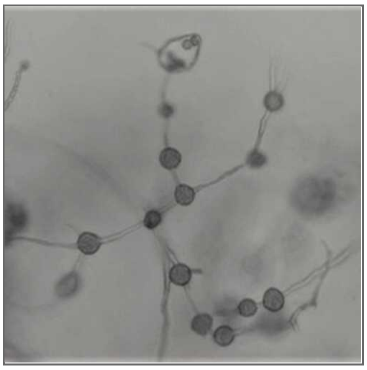

OCT 29, 2023

Phytophthora pluvialis culture cultivation, maintenance, and detached needle assay protocol

Sophie Eccersall[1], Leann Seesom Vinson[1], Rebecca McDougal[2], Claudia-Nicole Meisrimler[1]

[1]School of Biological Science, University of Canterbury, Private Bag 4800, Christchurch 8041, New Zealand;
[2]Scion (New Zealand Forest Research Institute, Ltd.), Private Bag 3020, Rotorua 3010, New Zealand

Sophie Eccersall

OPEN ACCESS

DOI:
dx.doi.org/10.17504/protocols.io.kqdg3p9d7l25/v1

Protocol Citation: Sophie Eccersall, Leann Seesom Vinson, Rebecca McDougal, Claudia-Nicole Meisrimler 2023. Phytophthora pluvialis culture cultivation, maintenance, and detached needle assay protocol. **protocols.io** https://dx.doi.org/10.17504/protocols.io.kqdg3p9d7l25/v1

License: This is an open access protocol distributed under the terms of the Creative Commons Attribution License, which permits unrestricted use, distribution, and reproduction in any medium, provided the original author and source are credited

Protocol status: Working
We use this protocol and it's working

ABSTRACT

Phytophthora pluvialis is an oomycete that primarily infects *Pinus radiata* and *Pseudotsuga menziesii* causing the destructive foliar disease red needle cast (RNC). Recent observations show that *P. pluvialis* can also infect western hemlock inducing resinous cankers. High-throughput and reproducible infection assays are integral to find key information on tree health and oomycete pathogenicity. In this protocol, we describe the propagation and spore induction of *P. pluvialis,* followed by detached needle assays for verification and quantification of virulence of *P. pluvialis* in *P. radiata* needles. These needle assays can be employed for high-throughput screening of tree needles with diverse genetic backgrounds.
In downstream analysis, Quantitative PCR (qPCR) was utilised to assess relative gene expression, as exemplified by candidate RxLR effector protein PpR01. Additional techniques like RNA sequencing, metabolomics, and proteomics can be combined with needle assays and can offer comprehensive insights into *P. pluvialis* infection mechanisms

Created: Feb 23, 2022

Last Modified: Oct 29, 2023

PROTOCOL integer ID: 58620

Keywords: Phytophthora pluvialis, Pine, Pinus radiata, detached needle assay, red needle cast, qPCR, zoospores, infection assay, mycelium

Funders Acknowledgement:
Radiata Pine Breeding Company

GUIDELINES

*This protocol was developed using a single isolate of *Phytophthora pluvialis* isolate (NZFS 4015) although other isolates have been trialed. It is known that some variation can exist between isolates, especially if they have been passaged in the lab over a long period. It can be beneficial to passage through pine needles or apple/pear prior to prepping for zoospore production to re-invigorate isolates every three to four months. The use of freshly isolated strains from the field can also be helpful for zoospore production.
This protocol can also be used to induce zoospore release in *Phytophthora cinnamomi.*

MATERIALS

Materials and reagents

1. Parafilm (PM-996)
2. Petri dishes (100 mm x 19.5 mm)
3. 70% ethanol
4. Pipette tips (1000 µL)
5. Foil (Standard aluminium foil)
6. Waste beakers
7. Sterilised pond water (untreated/unchlorinated water)
8. Sterilised Deionised water (SDW)
9. 20% Clarified V8 agar (cV8)
10. 20% cV8 broth
11. *Phytophthora pluvialis* isolates*
12. *P. radiata/Pseudotsuga menziesii/*other plant material for infection assays

Equipment and software

1. Laminar flow hood
2. Scalpel
3. Tweezers
4. Haemocytometer (Marienfeld Superior, Neubauer- improved cell counting chamber)
5. Bunsen burner
6. Lamp (6 x LED 3W MAX bulb)
7. Light microscope (Nikon SE Stereo Ocular Light Microscope, but any light microscope can be used)
8. Scanner (Epson Perfection V700 Photo with Dual Lens System)
9. Fridge (4 °C)
10. Incubator (17-20 °C), no light required
11. Climate controlled incubator with light (Contherm Biosyn Incubator 6000CP)
12. Software: ImageJ
13. Qubit™ 2 fluorometer or equivalent
14. Qubit™ RNA BR Assay Kit or equivalent
15. Thermofisher™ PowerTrack™ SYBR Green Master Mix or equivalent

16. Qiagen Rotor-Gene Q qPCR and accompanying software or equivalent

Primer sequences for qPCR

A	B	C
Primer	Forward sequence (5’-3’)	Reverse sequence (5’-3’)
PPR01-1	AAGCATTCCCCCGTATCACG	TCTTCGTCGTCCTTGGCATC
PACT-1	CGACCTGCGCCTTAGTGAAA	GCCCATTTCGTCGGTTTGAG

Media used in this protocol:

20% cV8 agar recipe (Modified from Jeffers, 2006)

Clarified and buffered V8 juice.

To buffer: Add 1.0 g of $CaCO_3$ for every 100 mL of V8 juice; mix thoroughly by magnetic stirrer

To clarify: Run V8 juice through multiple layers of sterilised cheesecloth; store aliquots in the freezer to maintain freshness

For 1 litre **20% cV8** agar:

●200 mL clarified/buffered V8 juice

●15 g agar

●800 mL distilled water

●30 mg beta-sitosterol*

*Dissolve beta-sitosterol in ethanol and gently warm before adding to mix. Add beta-sitosterol while stirring with a magnetic stirrer. Beta-sitosterol will precipitate in the cV8 media but should dissolve back out during autoclaving. Give a vigorous shake if necessary.

20% cV8 broth recipe

Same process as agar recipe, minus agar powder. Autoclave.

Antibiotic cV8 agar

Same process as cV8 agar recipe, with the addition of rifampicin (10 mg/mL), carbenicillin (100 mg/mL), nystatin (25mg/mL)

BEFORE START INSTRUCTIONS

All steps and procedure of the protocol should be completed in laminar flow hood wherever possible

A. Growth of *P. pluvialis* from existing culture plates

1 Grow *P. pluvialis* isolate on 20% cV8 agar (modified from Jeffers, 2006) at ~18 ˚C.

2 Sterilise tweezers with 70% ethanol and flame.

3 Subculture existing culture onto fresh cV8 agar plates using the wide end of a pipette tip to create three plugs, and place in a triangular position onto fresh cV8 agar plates with tweezers (see figure 1).

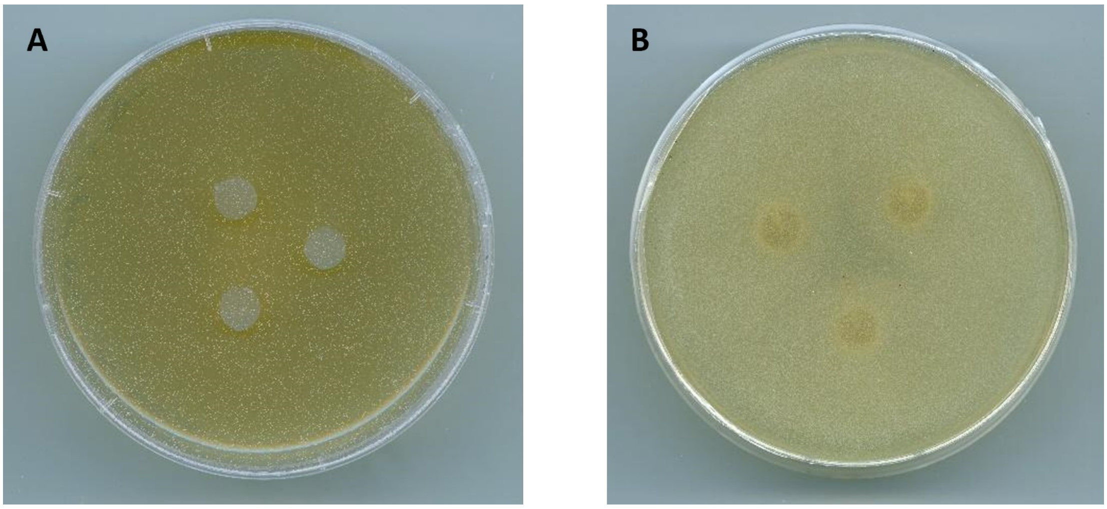

Figure 1. A. Plugs of mycelium from an existing plate placed onto fresh cV8 agar. B. *P. pluvialis* mycelial growth on cV8 agar after two weeks. *P. pluvialis* has appressed hyphae phenotype with an angular and petaloid growth.

4 Seal plates with parafilm, and store upside down in an incubator at 17-21 ˚C in the dark. *For zoospore production, allow plates to grow for 7-10 days, or until mycelium leading edge has filled the plate. Once mycelium has filled the plate, begin the replating procedure for maintenance.*

B. Zoospore preparation

5 Sterilise tweezers and scalpel with ethanol and flame.

6 Cut ten 1cm×1cm cubes of mycelium grown on a cV8 agar plate (fully grown, 7-10 days) and place into petri dishes containing ~15 mL sterilised cV8 broth. Make sure all cubes are evenly dispersed and floating in broth (see figure 2A).

7 Seal plate in parafilm, and store in 17-21 ˚C in the dark for two days. Mycelium starts to grow beyond the cut edge of the agar, floating in the cV8 broth (see figure 2A). P. pluvialis *isolate 4015 grows the best at 21˚C, but anything in the range of 17-21 °C works well).*

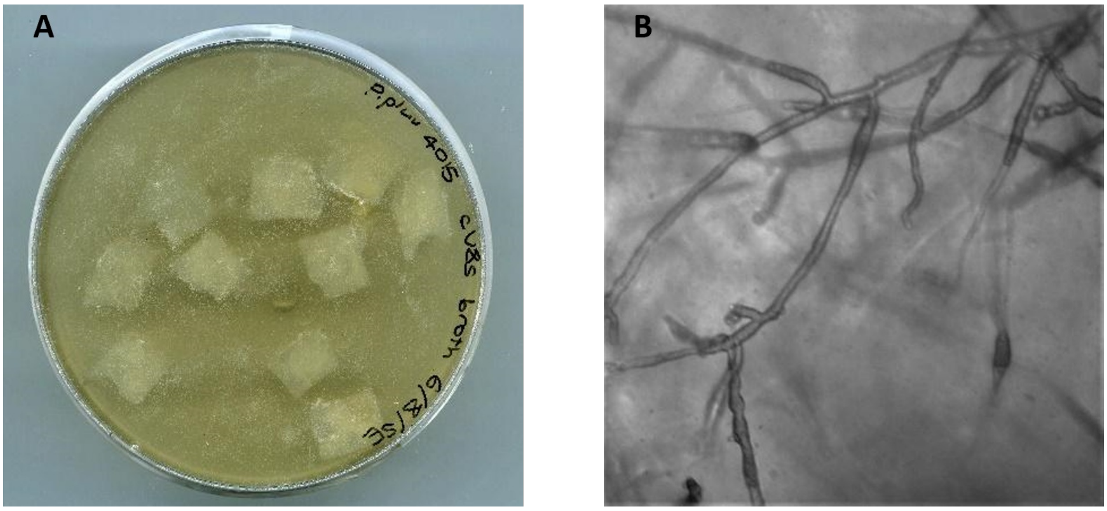

Figure 2. A. *P. pluvialis* agar cubes in cV8 broth after two days. You will notice the new mycelial growth past the edges of the cube. B. View of mycelium under 20× magnification lens.

Zoospore preparation step 2, day three

8 Remove broth as thoroughly as possible from plates.

9 Rinse multiple times with SDW to ensure all traces of broth have been removed. *This step is critical. The next step is starving the* Phytophthora *of essential nutrients to encourage the growth of sporangia.*

10 Soak mycelial mats in SDW for an hour. The mats should be pale pink/orange in colour.

11 Remove SDW ensuring all traces of calcium carbonate from the broth have been removed, and replaced with ~15 mL of sterile pondwater (see figure 3A).

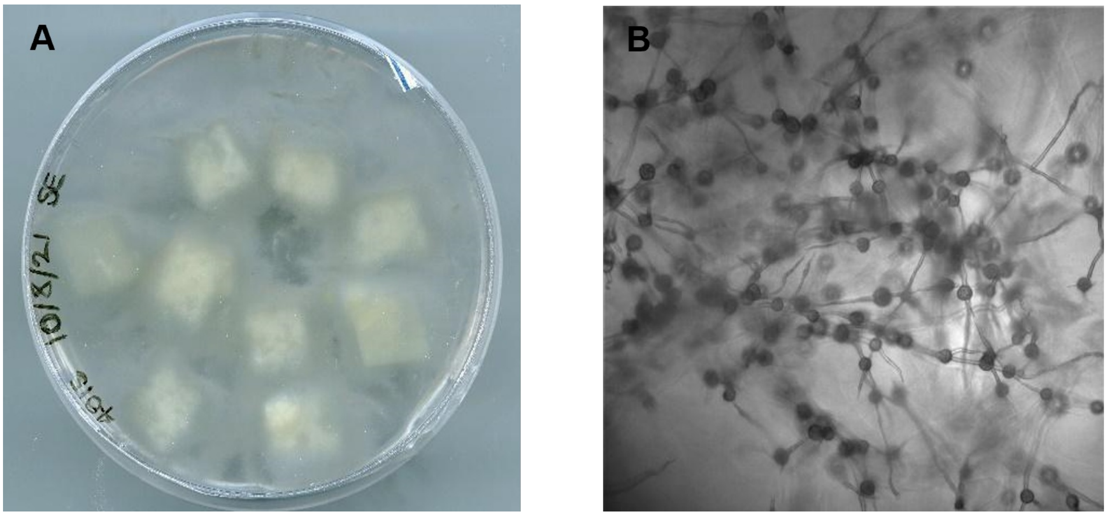

Figure 3. A. Plates flooded with sterile pond water. Mycelial cubes appear very pale orange/pink and all cV8 broth was removed. B. Hyphal swelling and immature sporangia developing in water; viewed under Nikon SE Stereo Light Microscope with 10× magnification.

12 Seal plates in parafilm, and store at 17-21 ˚C for 2-3 days in the dark (depending on the appearance of sporangia, see figure 3). *Immature sporangia are spherical in shape and will not release zoospores (see figure 3B). Mature sporangia have a characteristic semi papillate, ovoid shape (see figure 4A).*

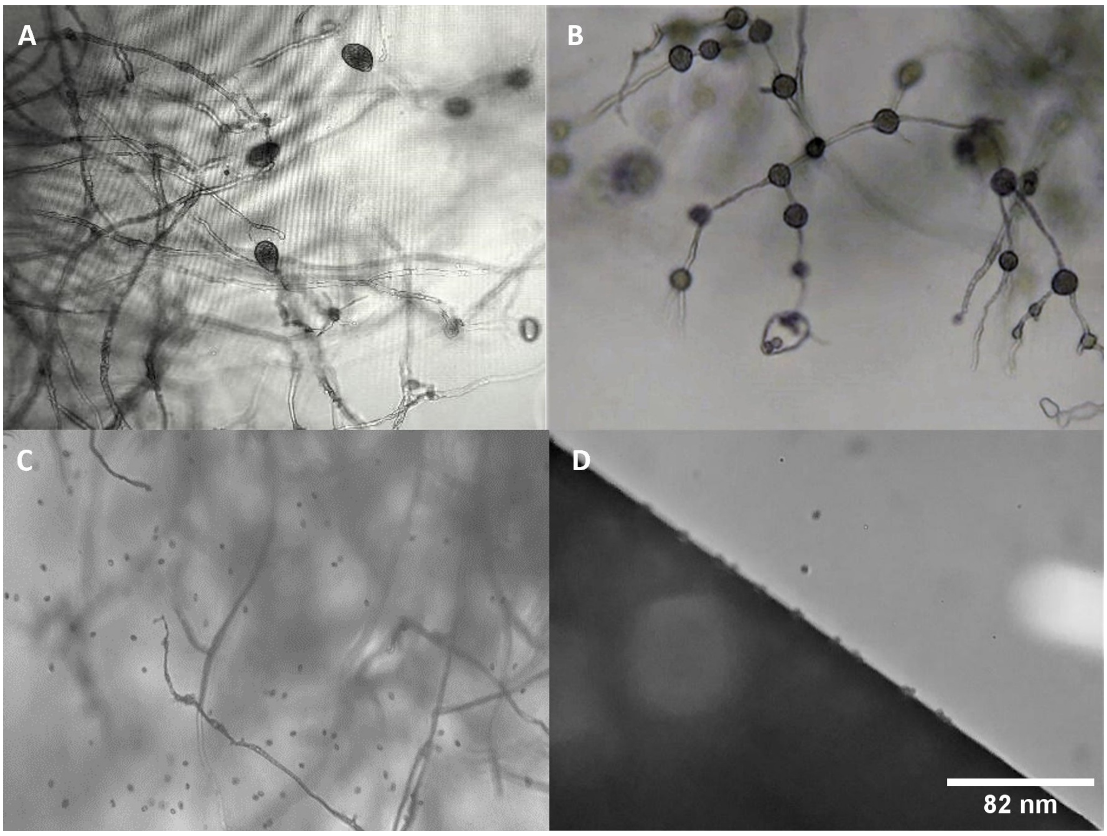

Figure 4. Sporangia and zoospores viewed under Nikon SE Stereo Light Microscope 20× magnification lens. A. Mature sporangia ready for release of zoospores; B. Zoospores mid-release; C. Released zoospores in solution; D. Zoospores aggregating on the surface of a needle.

Zoospore production step 3, day six/seven

13 If mature sporangia are present (see figure 4A, B), place plates into a 4 ˚C fridge for at least one hour at minimum. Plates must be cold to the touch.

14 Place under bright light for at least five minutes.

15 Place under microscope at 10× magnification to observe zoospore release (see video 1 & 2).

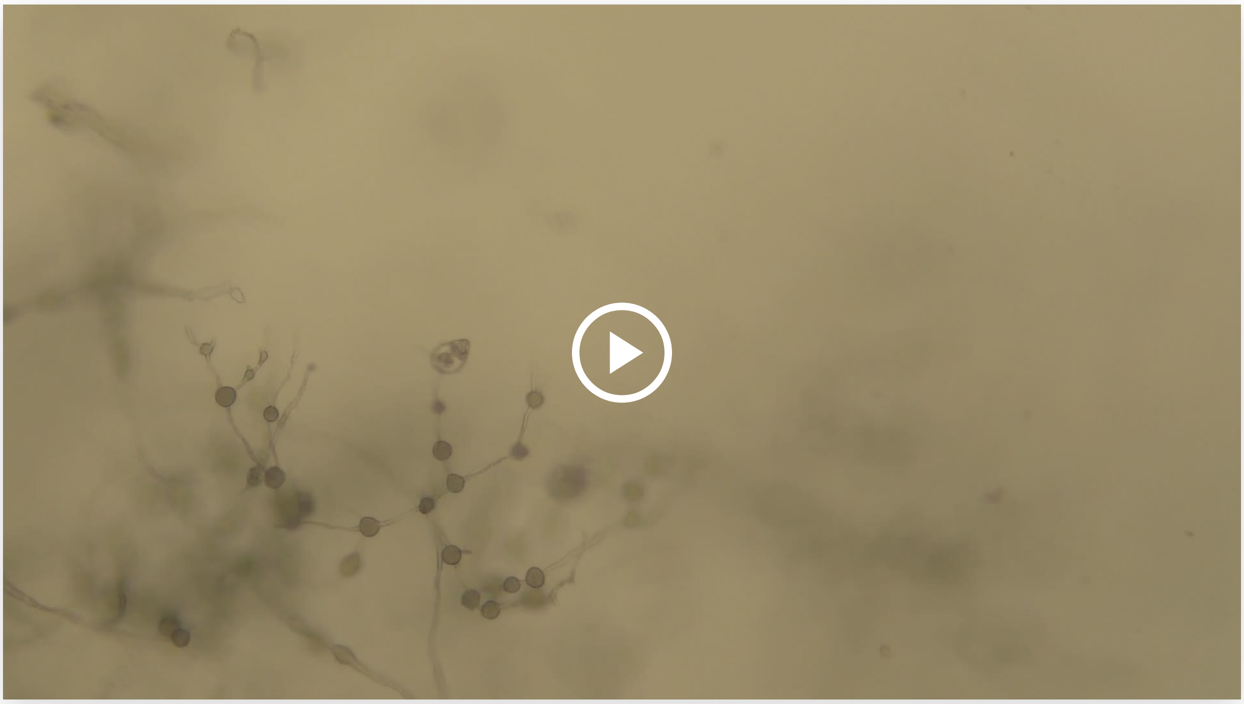

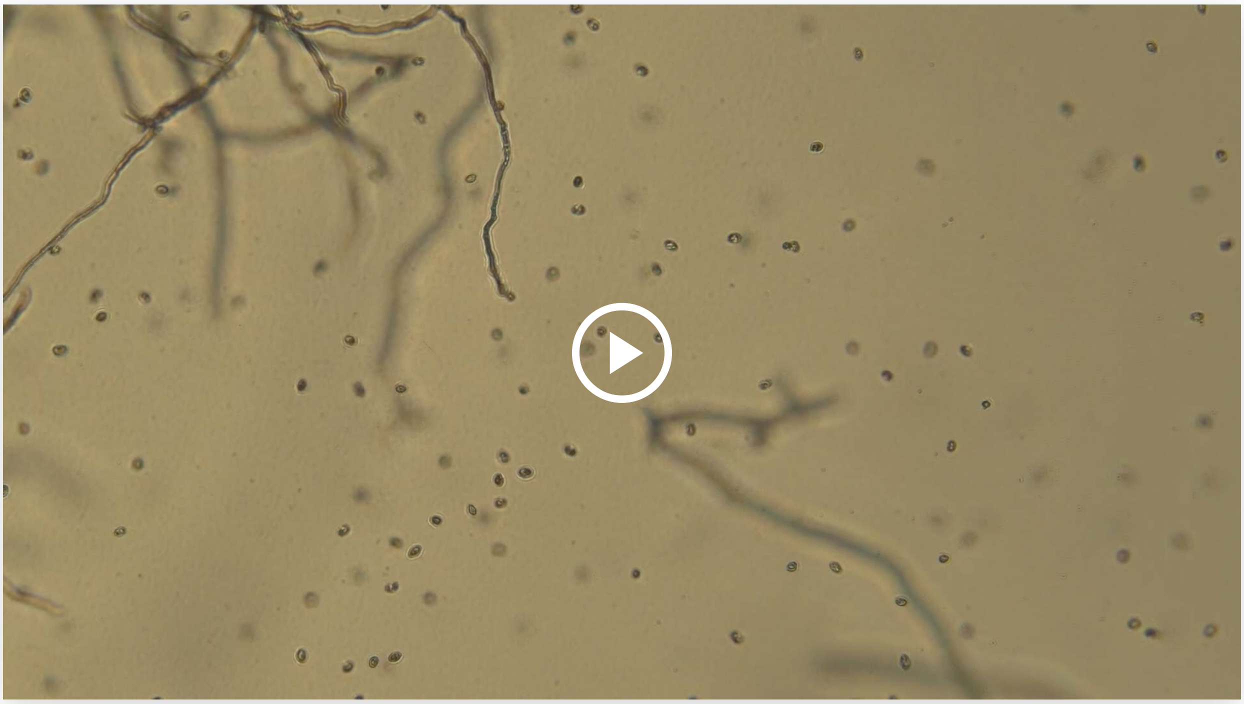

16 If no zoospores have been released, place back under the light for intervals of 10 minutes, followed by viewing under the light microscope (10× lens) to check for release.

17 If zoospores have still not released, repeat chilling and light treatment. If no release occurs after 30 min, leave plates for another half to full day in the fridge (plates can be left in the fridge overnight).

18 If zoospore release is successful, prepare needle assays. *P. pluvialis* zoospores have a short life span of ~2 hours, use quickly.

19 Once zoospore solution has been siphoned off, plates can be reinvigorated with cold sterile pondwater and light treatment to release a second and third wave of zoospores.

20 Use a haemocytometer (Marienfeld Superior, Neubauer- improved cell counting chamber) and 10-20 µL of zoospore solution to count spores under a light microscope at 10× magnification. Count at least 6 samples per plate and average. Aim for a working concentration of 5 x 10^3 zoospores/mL. This concentration can be adjusted as needed.

20.1 **Troubleshooting.** Release of zoospores is crucial to effective detached needle assays. If there is a lack of mature sporangia, place the plates with SPW and mycelial mats under a bright lamp for
24 hours. Check for the presence of mature sporangia. Following this, if there are still no mature sporangia, place the same plates into the fridge overnight, and then back under the lamp. Occasionally, this will need to be done several times to push the sporangia to mature and allow for zoospore release.

C. Detached Needle Assays

21 Harvest fresh pine needles (of any age, although best results are seen on needles of at least one year old).

22 Take 5 mL of zoospore solution and place into 50 mL falcon tubes. *Be gentle when handling the zoospore solution* - P. pluvialis *zoospores encyst very easily when knocked.*

23 Place needles fascicle end down into zoospore solution.

24 Seal, cover tubes in foil and leave overnight standing upright.

25 18-24 hours later, prepare petri dishes by layering 3-4 sterilised paper towels into a dish.

26 Thoroughly dampen the paper towels with sterile H_2O.

27 Place needles incubated with spores as flat as possible into the dishes, seal with parafilm (see figure 5).

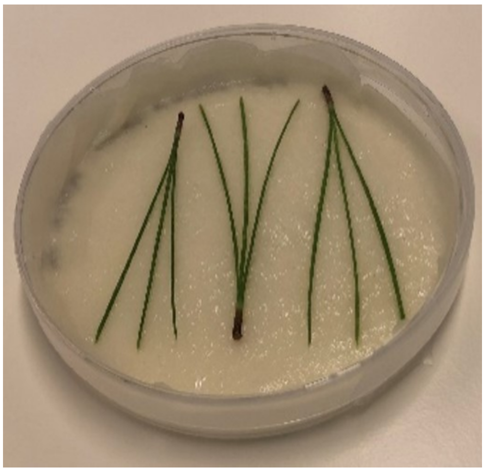

Figure 5. *P. pluvialis* infection assay on freshly detached needles.

C. Detached Needle Assays

28 Place into an incubator (temperature set at 17˚C; 12 hours daylight, 12 hours darkness), and check at 3 days after infection (DAI), 7 DAI and 12 DAI intervals (see figure 6).

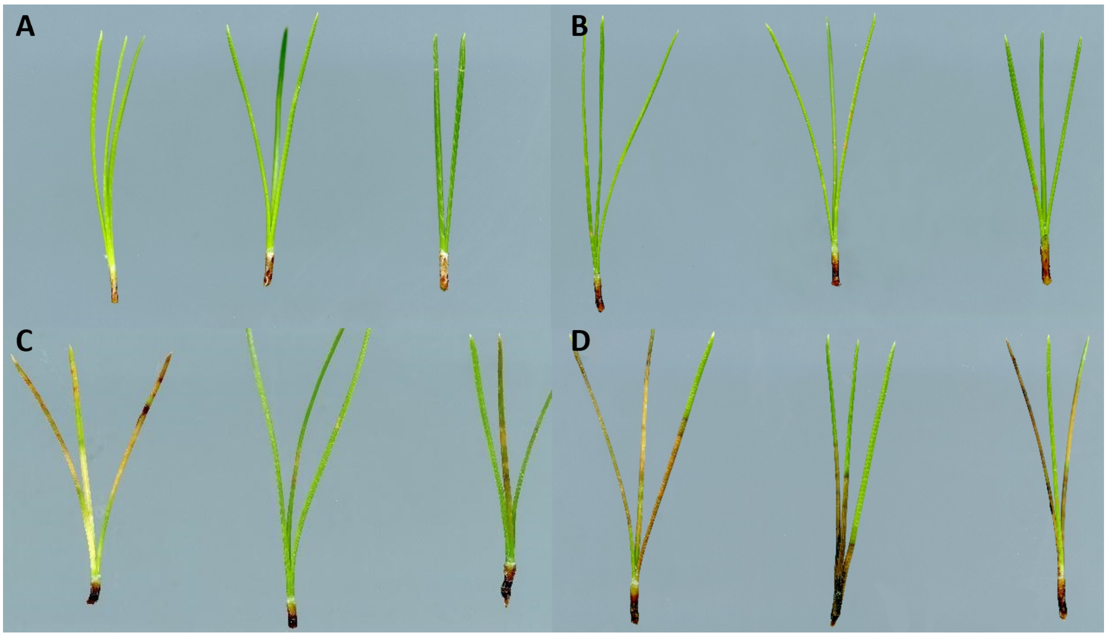

Figure 6. Infection process on mature *P. radiata* needles. A. Day zero. B. 3 DAI C. 7 DAI. D. 12 DAI.

D. Symptom monitoring and scoring

29 Measure and record the total number, length, and severity of lesions (see figure 6 and 7).

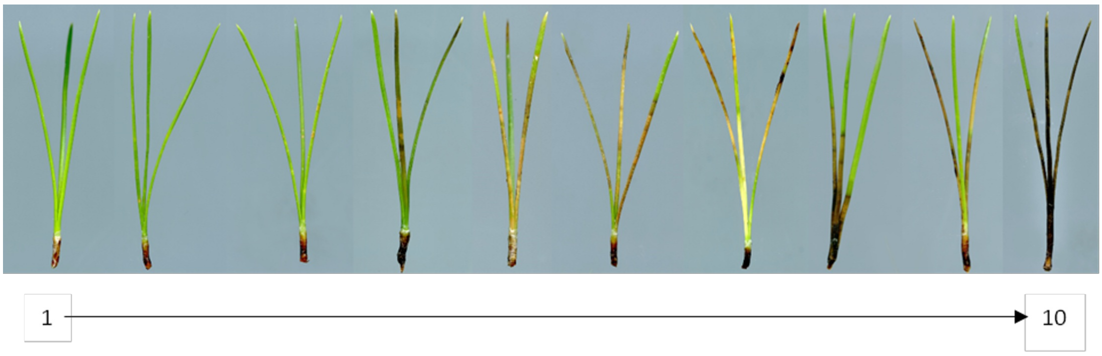

Figure 7. 1-10 scale of severity of RNC lesions on *P. radiata* needles.

30 Note that symptoms may appear differently depending on the age of the needles. Needles from young plantlets show more yellowing and less discrete olive bands.

E. Passaging Isolates

31 Take infected needles from assay plates or collected in the field from infected trees and rinse with 70% ethanol, follow by washing with sterile H_2O, repeat 3-times.

32 Pat dry with sterile paper towel and place on antibiotic cV8 agar plates (cV8 agar recipe, with the addition of rifampicin (10 mg/mL), carbenicillin (100 mg/mL), nystatin (25mg/mL)).

33 Allow mycelium to grow on plates, this can take up to 7 days (see figure 8).

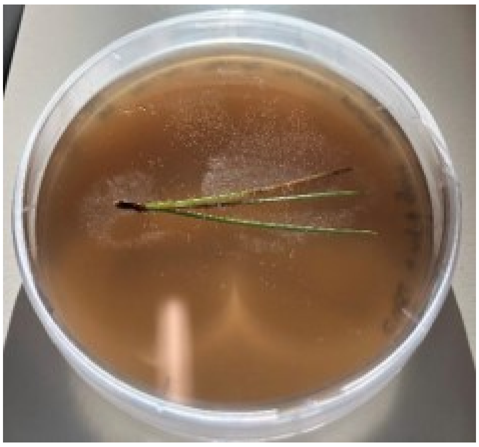

Figure 8. New mycelial growth from infected needle onto antibiotic plate.

34 As soon as possible, transfer plugs of mycelium onto new non-antibiotic cV8 plates.

34.1 Passaging isolates can be used for spore production. Approximately 21 days after infection, new sporangia are visible growing out from the lesions on the pine needle (see figure 9). These sporangia can also be used for zoospore release by placing them in cold SPW and placing them under bright light.

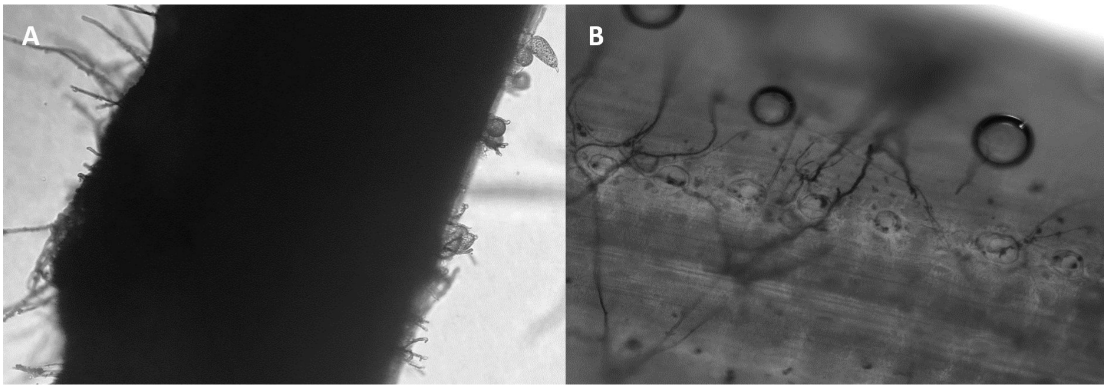

Figure 9. A. New mature sporangium growing on *P. radiata* needle 21 DAI. Viewed under Nikon SE Stereo Light Microscope 10× Lens. B. Mycelium coiling around the surface of needle and into stomata. Brightfield microscope images captured using a Zeiss AXIO506 microscope at 40× magnification.

Confirmation of infection via qPCR

35 Extract RNA from mycelium, non-infected and infected needle samples at the preferred time point using users RNA extraction kit of choice.

36 Measure extracted RNA concentration either via Quibit™ fluorometer (recommended) or equivalent.

37 Using the measured RNA concentrations, dilute all samples to 50 ng/µL.

38 Perform reverse transcription on the RNA samples using an appropriate kit of choice. You will need a cDNA concentration of 50 ng/µL for each sample.

39 Serially dilute the primers for the RxLR PpR01 and actin from 1:10^6.
For primer sequences, see materials section.

40 Perform the qPCR according to manufacturer's instructions. If using the Thermofisher™

PowerTrack™ SYBR Green Master Mix, use the following settings:

A	B	C	D
Step	Temperature	Time	Cycle
Enzyme activation	95°C	2 minutes	1
Denaturation	95°C	15 seconds	40
Extension	60°C	60 seconds	

41 Analyse qPCR results with the appropriate software.
